# Real-time monitoring of PtaHMGB activity in poplar transactivation assays

**DOI:** 10.1186/s13007-017-0199-x

**Published:** 2017-06-15

**Authors:** José M. Ramos-Sánchez, Paolo M. Triozzi, Alicia Moreno-Cortés, Daniel Conde, Mariano Perales, Isabel Allona

**Affiliations:** 1 Centro de Biotecnología y Genómica de Plantas, Universidad Politécnica de Madrid (UPM) - Instituto Nacional de Investigación y Tecnología Agraria y Alimentaria (INIA), Campus Montegancedo UPM, 28223 Pozuelo de Alarcón, Madrid, Spain; 2 Departamento de Biotecnología-Biología Vegetal, Escuela Técnica Superior Ingeniería Agronómica, Alimentaria y de Biosistemas, Universidad Politécnica de Madrid (UPM), 28040 Madrid, Spain

**Keywords:** Transcriptional reporter, Transactivation assay, Chromatin remodeling, High mobility group, Diurnal and circadian rhythms, Poplar, *Populus tremula* *×* *alba*, Transient expression, LHY2

## Abstract

**Background:**

Precise control of gene expression is essential to synchronize plant development with the environment. In perennial plants, transcriptional regulation remains poorly understood, mainly due to the long time required to perform functional studies. Transcriptional reporters based on luciferase have been useful to study circadian and diurnal regulation of gene expression, both by transcription factors and chromatin remodelers. The high mobility group proteins are considered transcriptional chaperones that also modify the chromatin architecture. They have been found in several species, presenting in some cases a circadian expression of their mRNA or protein.

**Results:**

Transactivation experiments have been shown as a powerful and fast method to obtain information about the potential role of transcription factors upon a certain reporter. We designed and validated a luciferase transcriptional reporter using the 5′ sequence upstream ATG of *Populus tremula* *×* *alba LHY2* gene. We showed the robustness of this reporter line under long day and continuous light conditions. Moreover, we confirmed that *pPtaLHY2::LUC* activity reproduces the accumulation of *PtaLHY2* mRNA. We performed transactivation studies by transient expression, using the reporter line as a genetic background, unraveling a new function of a high mobility group protein in poplar, which can activate the *PtaLHY2* promoter in a gate-dependent manner. We also showed PtaHMGB2/3 needs darkness to produce that activation and exhibits an active degradation after dawn, mediated by the 26S proteasome.

**Conclusions:**

We generated a stable luciferase reporter poplar line based on the circadian clock gene *PtaLHY2,* which can be used to investigate transcriptional regulation and signal transduction pathway. Using this reporter line as a genetic background, we established a methodology to rapidly assess potential regulators of diurnal and circadian rhythms. This tool allowed us to demonstrate that PtaHMGB2/3 promotes the transcriptional activation of our reporter in a gate-dependent manner. Moreover, we added new information about the PtaHMGB2/3 protein regulation along the day. This methodology can be easily adapted to other transcription factors and reporters.

**Electronic supplementary material:**

The online version of this article (doi:10.1186/s13007-017-0199-x) contains supplementary material, which is available to authorized users.

## Background

A precise synchronization of plant development with environmental changes largely depends on an accurate control of gene expression. Transcriptional regulation is being comprehensively studied in *Arabidopsis*, however, its understanding in poplar has lately emerged along with the successful application of the genetic transformation methods. Several laboratories have undertaken this research area by doing functional and molecular analysis of poplar transcription factor in wood development, branching, nitrogen acquisition, flowering, and growth-dormancy cycles [1–16]. Nevertheless, the time required for functional characterization in perennial trees is usually a great limitation. A rapid assessment of the potential effect that a protein or a protein family could carry out upon transcription on its putative target genes, will accelerate the acquisition of knowledge concerning regulation of gene expression in poplar.

Transcriptional reporters are essential tools for spatio-temporal gene expression studies in plants [17–21]. They consist of a transcriptional fusion of a reporter gene, e.g. β-glucoronidase (GUS) [22], green fluorescent protein (GFP) [23], or luciferase (LUC) [24], and the *cis* regulatory and promoter sequences of a given gene under study. Luciferase-based transcriptional reporters have been successfully applied to study regulation of diurnal and circadian gene expression in plants [10, 17, 24–31]. The rapid degradation of the LUC protein in vivo allows a real-time monitoring of gene transcription in 24 h time course experiments [24]. Thus, real-time bioluminescent assays have been shown to robustly reproduce the mRNA expression pattern [24]. In addition to this, luciferase-based transcriptional reporters have been used to identify new regulators of the circadian clock by mutagenesis screening [25], or by functional studies with a candidate gene [10, 32, 33]. Even more, they have also been useful for the analysis of signal transduction pathways operating upstream and downstream the clock [30, 34–36], and to assess the contribution of chromatin dynamics to circadian rhythms by chemical inhibition of histone deacetylation pathway [17].

High mobility group (HMG) proteins were identified as part of non-histone nuclear protein extract from calf thymus [37]. All HMG proteins present a non-sequence-specific DNA binding domain (HMG-box), besides additional structural features that allow their separation in three different families: HMGA, HMGB and HMGN [38, 39], being the HMGB type the most studied in plants. This latest subgroup presents a basic N-terminal tail and an acidic C-terminal tail, which interaction regulates the protein affinity to the DNA [40]. Moreover, that tails also contain information for protein localization [41], but the deciphering of this code it is being elusive.

An interesting feature of some HMG proteins is that their mRNA or protein present diurnal oscillations. For instance, Hmgb1 protein has a circadian regulation in rat retinal photoreceptor cells [42]. In addition, HMG1 but not HMG2 mRNA from *Pharbitis nil* is controlled by an endogenous rhythm [43, 44], revealing a gene specificity regarding this kind of regulation. Nevertheless, whether this control has a feedback in the regulation of the circadian clock or upon any of its genes has not been explored so far.

HMG proteins alter the architecture of the chromatin, facilitating the histone 1 eviction, and then relaxing the chromatin [45, 46]. To this aim, the acidic tail, together with the HMG-box, interacts with DNA, while basic residues interact with histones, loosening the nucleosome [45, 47]. These proteins are also involved in the recruitment and stabilization of transcription factors to their binding sites [45, 48–51]. Due to these functions, HMG proteins have also been described as chromatin chaperones serving as transcriptional facilitators [52, 53].

Post-translational modifications such as methylation, acetylation and phosphorylation play an important role in HMG function [54]. For instance, those modifications are involved in nuclear export of HMGB1 in mouse [55–57] and in nucleosome interaction and subcellular distribution during mitosis for human HMGN1 [58–61]. In maize, phosphorylation of HMGB1 and HMGB2/3 reduce their affinity by DNA [62]. This reduction is primarily caused by the stabilization of the interaction between the acidic and basic tails of these proteins [40]. Moreover, this phosphorylation also reduces the interaction between HMGB proteins and transcription factors [48], but has no role in protein localization [41].

In this article, we presented a step-by-step procedure to generate and validate a stable luciferase-based transcriptional reporter on the hybrid poplar (*Populus tremula* *×* *P. alba*) clock gene *LHY2*. Moreover, we adapted to poplar an automatic luminescence microplate reader protocol to monitor diurnal and circadian rhythms of the *LHY2* reporter in real-time during several days. Furthermore, we established a rapid transactivation assay to determine the potential function that a protein or a protein family has under different light conditions, in a temporal manner and over a certain reporter. Using this methodology, we were able to analyze the transcriptional activity of two members of poplar HMGB family. Our results showed a dark-dependent transcriptional activity of PtaHMGB2/3. Additionally, we provided information about the posttranslational control of this member of HMGB protein family.

## Methods

### Plants, strains and growth conditions

For gene expression analysis and genetic transformation, we used *P. tremula* *×* *alba* INRA clone 717 1B4. For gene expression analysis, poplars were cultured in vitro and transferred to 3.5-L pots containing blond peat as we previously described in [7]. Poplar leaf samples were harvested under different light conditions. Plants were grown under long days (LD) 16 h:8 h light–dark at 21 °C. From LD, plants were subsequently shifted to continuous light (LL) or to continuous dark (DD), at 21 °C, during 48 h.

For transient expression experiments in poplar, *pPtaLHY2::LUC* poplars were grown in vitro in a Murashige and Skoog medium modification 1B (pH 5.7) supplemented with 2% sucrose and with indole acetic and indole butyric acids (0.5 mg/L) containing 0.7% (w/v) plant agar under LD at 21 °C during 3–4 weeks.

For transient expression experiments in *Nicotiana benthamiana,* plants were grown in greenhouse from seeds under 16 h:8 h light–dark cycles at 21 °C during 3–4 weeks.

We used *Escherichia coli* DH5α strain for gene cloning and *Agrobacterium tumefaciens* GV3101/pMP90 strain [63] for transient expression and plant transformation assays.

### Plasmid constructs

The selected promoter sequence of *PtaLHY2* was amplified from *P. tremula* *×* *alba* genomic DNA using specific primers (Additional file 1: Table S1). For PCR, Pfu Ultra Hotstart High-Fidelity DNA Polymerase (Agilent, Santa Clara, CA, USA) was used and the PCR fragments were purified and cloned into pCR-Zero-Blunt vector (Invitrogen, Carlsbad, CA, USA). A second PCR was carried out for amplifying the promoter with Gateway tails, using pCR-Zero-Blunt-pPtaLHY2 as a template. The amplified fragment was purified and cloned into pDONR207 Gateway vector. This Gateway cassette was transferred into the destination binary vector LucTrap-3(GW) [64], generating the final *pPtaLHY2::LUC* construct.

We used Golden Braid (GB) technology [65] to generate overexpression and knock-down constructs in this work. The GB_parts used in this study are detailed in the Additional file 2: Table S2.

For functional studies, *PtaHMGB2/3* and *PtaHMGB6* were amplified from *P. tremula* *×* *alba* cDNA samples. Fragments were purified and cloned in a pUPD vector. Then, we generated a complete transcriptional unit creating *35S::PtaHMGB2/3::tNOS* and *35S::HMGB6:tNOS* in 1alpha2 expression vector, respectively. Afterwards, each of these constructs, were cloned in a 1omega1 expression vector together with *1alpha1_35S::GFP::tNOS* to select the successfully infiltrated leaf areas in the transient expression assays. For knock-down construct, HMGB2/3 artificial microRNA was generated as described in [66] and adapted to GB. Then, it was cloned in a 1omega1 together with *35S::GFP::tNOS.*

### Stable and transient plant transformation

Hybrid poplars were transformed with a binary plasmid carrying the *pPtaLHY2::LUC* construct, via an Agrobacterium-mediated protocol previously described [67].

Transient poplar transformation was performed following the protocol previously reported [68]. Transient *N. benthamiana* agroinfiltration experiments were done as previously described [69]. *N. benthamiana* leaves samples were harvested 3 days post infiltration for protein assays and fluorescence microscopy.

### Luciferase reporter assays

We screened the transgenic lines of *pPtaLHY2::LUC* by spraying these plants with d-luciferin 5 mM and then measuring bioluminescence at ZT0 using a CCD camera (NightOwl–Berthold), exposing the plants during 15 min. Three out of 12 lines were selected to further work.

To perform continue monitoring luciferase activity, we used leaf tissue from 3 to 4 weeks old plants carrying the *pPtaLHY2::LUC* transcriptional reporter. The leaf discs were done using a hole puncher leaving a total area of 32 mm^2^ approximately. To avoid dehydration of the tissue while cutting the discs, leaves were placed over a filter paper previously dampened with water. After this, they were left in a 96-well plate with Murashige and Skoog 1B medium (pH 5.7) without sucrose. Fifty microlitres of d-luciferin 5 mM were added into every well on top of the leaf disc. A qPCR film was used to cover the plate. We made three holes in the plastic seal covering every well to avoid condensation with a syringe needle (BD Microlance 3–30F ½″ 0.3 × 13 mm). The TriStar2 LB 942 luminometer (Berthold Technologies, Bad Wildbad, Germany) was used to register the luciferase activity measuring 5 s per well every 3 h. Additional file 3: Figure S1 illustrates the different steps of the microplate preparation for luminescence measurement.

We did plots and statistical analysis using RStudio [70]. To infer the period and the relative amplitude error we use Spectrum Resampling application [71].

### Phylogenomic and phylogeny studies

The phylogenomic comparison among the different 5′ *cis* regulatory sequences were done using mVISTA online application [72].

We used protein sequences to do phylogenetic trees. We used MUSCLE algorithm to do the alignments (200 iterations) and neighbour-joining, UPGMA and maximum likelihood to infer the tree. To carry out this work we used MEGA7 application [73].

### RT-PCR expression analyses

The samples analyzed in these experiments were pools of at least three plants. Total RNA extraction was done using NucleoSpin RNA Plant kit (Macherey-Nagel, Düren, Germany). Single-stranded cDNA synthesis, primer design and data analysis were performed as described previously [10, 74–76]. Real-time PCR were carried out using LightCycler 480 II (Roche, Basel, Switzerland) following the manufacture procedures.

### Microscopic studies

For protein subcellular localization, transiently expressing PtaHMGB2/3:YFP and YFP *N. benthamiana* leaves were sectioned and observed using Zeiss Axiophot epifluorescence microscope. Images were captured with 40× magnification by a Leica DFC 300FX CCD color camera equipped with Leica Application Suite 2.8.1 build 1554 acquisition.

For the quantification of YFP, HMGB2/3:YFP and HMGB6:YFP fluorescence, discs images were captured by a Leica MZ10F fluorescence binocular loupe, using 10× magnification. Images were recorded by a Leica DFC 300FX CCD color camera equipped with Leica Application Suite 2.8.1 build 1554 acquisition. Relative fluorescence (fold-increase) was calculated using Fiji software [77] as previously described [78].

### Protein assays

The *35S::3xHA:HMGB2/3:tNOS* construct was transiently expressed in *N. benthamiana* leaves. After 3 days post infiltration, leaf samples were collected at the indicated times point in each experiment.

For *3xHA:HMGB2/3* immunodetection, leaf samples were ground and 0.25 g of tissue were extracted with RIPA protein extraction buffer (50 mM Tris–HCl pH 8.0, 150 mM NaCl, 1%Triton X-100, 0.5% sodium deoxycholate, 0.1% SDS). Protein extracts were clarified by centrifugation at 13,000 rpm for 1 min at 4 °C. Total protein concentration was quantified using Bradford protein assay [79]. Afterwards 100 µg of total protein sample was loaded in SDS-PAGE.

For cell-free degradation assays, leaf samples were ground and 0.25 g of tissue were extracted with cell-free buffer (50 mM Tris–HCl pH 7.5, 100 mM NaCl, 10 mM MgCl_2_, 5 mM DTT, 5 mM ATP). Protein extracts were clarified by centrifugation at 13,000 rpm for 1 min at 4 °C. Equal amounts of extracts were transferred to individual tubes and incubated at 30 °C or left on ice for 90 min. Reactions were stopped by adding protein gel-loading buffer and loaded in SDS-PAGE.

To test the implication of the proteasome, we repeated the previous cell-free degradation assay with samples harvested at ZT6. Samples incubated at 30 °C were supplemented with MG132 50 μM or DMSO as a control. Reactions were stopped by adding protein gel-loading buffer and loaded in SDS-PAGE.

### Western blot

Western blot analysis was performed as previously described [33]. Anti-HA peroxidase-conjugated antibody (Sigma, St. Louis, MO, USA) was used in 1:1000 dilution.

## Results

### Generation of *pPtaLHY2::LUC* transcriptional reporter to monitor diurnal and circadian rhythms in poplar

**Fig. 1:**
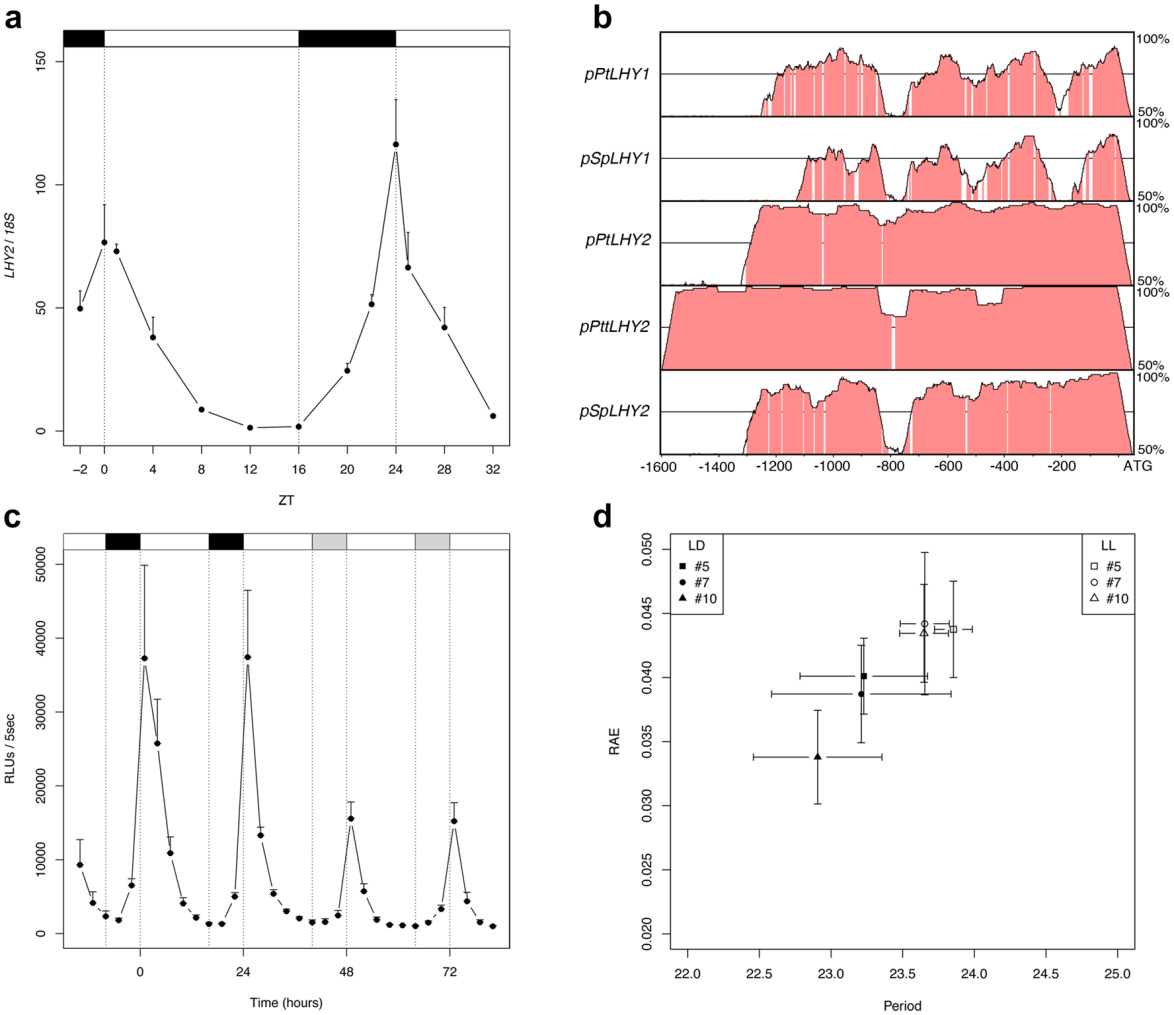
Design and validation of *pPtaLHY2::LUC* transcriptional reporter in poplar. **a** *PtaLHY2* mRNA expression profile in long day (LD) conditions obtained by qRT-PCR. Data indicates mean ± SD of three technical replicates. This experiment was performed twice. A representative result is shown in *this figure*. *Upper bar* indicates the photoperiod: *black boxes* represent the night and *white boxes* represent the day, respectively. **b** Phylogenomic analysis obtained by mVISTA plot using the *LHY1* and *LHY2* 5′ upstream ATG sequences from *Populus trichocarpa* (Pt), *Populus tremula* × *tremuloides* (Ptt) and *Salix purpurea* (Sp). *Populus tremula* × *alba* was used as a reference sequence. Regions of 6 bp with more than 70% of identity are shadowed in pink. **c** *pPtaLHY2::LUC* activity detected by luminescence assay during 2 days under LD and 2 days under continuous light (LL) conditions. The activity of *pPtaLHY2::LUC* line #5 is shown in *this figure*. Data indicates mean of three independent assays. *Error bars* indicate SD of the mean. **d** Period versus relative amplitude error analysis for the three transgenic lines analyzed. Data indicate mean of three independent assays for every line. *Error bars* indicate SD of the mean

We wanted to select a transcriptional reporter capable to monitor diurnal gene expression in a temporal manner, in the hybrid *P. tremula* *×* *alba* adapted to our geographical region. To this aim, we chose the *LATE ELONGATED HYPOCOTIL* (*LHY*) gene, which is one of the circadian clock central genes. LHY is directly linked to the regulation of developmental processes such as, hypocotyl growth and flowering in *Arabidopsis* [80, 81] and winter dormancy in poplar [10]. *Populus* has duplicated *LHY* genes, designated *LHY1* and *LHY2*, showing conserved daily expression patterns [10, 82]. *LHY2* expression is higher than *LHY1* in *Populus* species, suggesting that *LHY2* may play a major role in the clock [10, 82]. Moreover, *LHY2* has already been used as a transcriptional reporter in the *P. tremula* *×* *tremuloides* hybrid poplar [68]. Firstly, we performed a phylogenetic analysis to identify *P. tremula* *×* *alba LHY2* using LHY protein sequences from *Populus* and *Salix* species (Additional file 4: Figure S2a). Hence, the phylogeny indicated that the LHY sequence encoded by Potri.014G106800 is PtaLHY2. To study the daily pattern of gene expression of *PtaLHY2*, we carried out time series qRT-PCR analyses of poplar mRNA from leaf samples collected in long day conditions (LD) (Fig. 1a). A well-marked oscillatory pattern was detected for *PtaLHY2* mRNA accumulation showing a peak at dawn, similarly to the diurnal expression reported for its poplar homologs *PnLHY2* and *PttLHY2*, and for *Arabidopsis LHY* [10, 82, 83].

It has been shown that photoperiodic and circadian *cis*-regulatory elements are conserved across the species and localized at the 5′ regulatory region of those genes [84]. Therefore, to identify potential *cis*-regulatory elements of *PtaLHY2,* we carried out phylogenomic comparison among the 5′ 1600 bp sequences of three poplar homologs and one willow species, upstream the translation start site (Fig. 1b). All 5′ sequences of *LHY2* showed higher similarity with the reference one (*P. tremula* *×* *alba*) than *LHY1*, even in the case of *Salix purpurea* (Fig. 1b). Due to the conservation observed in all the alignments, we selected the 1300 bp of 5′ *PtaLHY2* sequence to create the binary vector *pPtaLHY2::LUC.* Using this construct we generated a stable transgenic poplar line using an *Agrobacterium*-mediated protocol. Individual lines were screened by bioluminescence analysis using a CCD camera, selecting three lines from a total number of 12 that showed high luciferase activity (#5, #7 and #10). We adapted and standardized to poplar samples a microplate reader system for monitoring luciferase activity in a temporal manner [33]. Leaf discs of poplar reporter lines #5, #7 and #10 were placed on 96-well plate in presence of luciferin 5 mM. The luminescence was recorded every 3 h under LD and continuous light (LL) conditions during 4 days (2 days in LD followed by 2 days in LL) (Fig. 1c; Additional file 4: Figure S2b, c). The results corroborate that our reporter reproduce the *PtaLHY2* expression pattern observed in LD and LL (Fig. 1a; Additional file 4: Figure S2d). Moreover, the period and relative amplitude error (RAE) of the three lines were calculated in both mentioned conditions. Mutations that affect the clock, usually present RAE values around 0.3–0.6 in reporter gene assays [29]. In our analyses, RAE was less than 0.05 in both conditions tested, confirming the robustness of the rhythms of our reporter lines [85]. Regarding the period, it presented a value around 24 h, the correct data for circadian clock genes. Interestingly, the period in LL was a bit higher than in LD, a situation usually observed in these transcriptional reporters in constant light conditions [17, 86] (Fig. 1d).

### Variation of *PtaHMGB2/3* but not of *PtaHMGB6* level affects the expression of *pPtaLHY2::LUC*

**Fig. 2:**
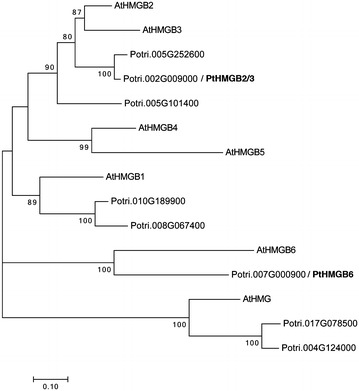
Molecular phylogenetic analysis of HMGB family by neighbour-joining method. The HMGB evolutionary history was inferred using the neighbor-joining method. The optimal tree with the sum of branch length = 4.147 is shown. The percentage of replicate trees in which the associated taxa clustered together in the bootstrap test (500 replicates) is shown next to the branches. The tree is drawn to scale, with branch lengths in the same units as those of the evolutionary distances used to infer the phylogenetic tree. The evolutionary distances were computed using the Poisson correction method and are in the units of the number of amino acid substitutions per site. The analysis involved 15 protein sequences. All positions containing gaps and missing data were eliminated. There were a total of 113 positions in the final dataset. The Potri.002G009000 and Potri.007G000900 were named as PtHMGB2/3 and PtHMGB6, respectively

HMGB proteins have a critical role in the regulation of gene expression acting as chromatin remodelers at nucleosome level and as chaperones upstream of specific transcription factors [52]. HMGB protein family presents several members that have not been previously characterized in poplar. We carried out a phylogenetic study using *Arabidopsis thaliana* and putative *Populus trichocarpa* HMGB proteins. We inferred the phylogeny using Neighbour-Joining (Fig. 2), Maximum Likelihood (Additional file 5: Figure S3a) and UPGMA (Additional file 5: Figure S3b) methods. We established well-supported homologies for all HMGB proteins except for AtHMGB4 and AtHMGB5.

To test the ability of these proteins to modify the expression of our transcriptional reporter, we selected two poplar proteins named as PtaHMGB2/3 and PtaHMGB6 (Fig. 2) for two reasons: (1) their divergence was sufficiently supported with high bootstrap values in the three analyses and (2) they have mostly conserved their HMG-box domain, but C-terminal and N-terminal domain show highly divergent sequences (Additional file 6: Figure S4).

**Fig. 3:**
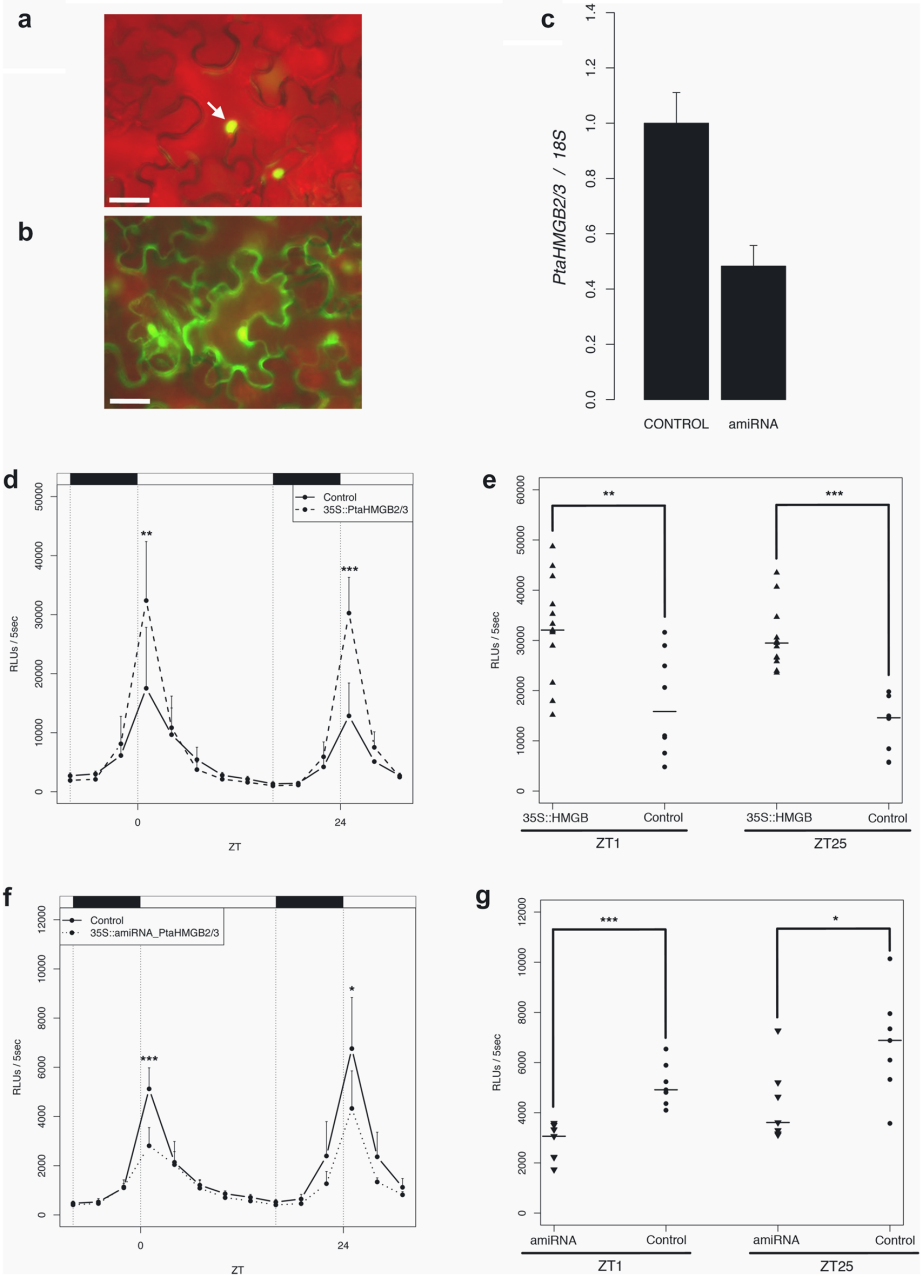
PtaHMGB2/3 activates *pPtaLHY2::LUC* reporter in a gate-dependent manner. **a**, **b** Fluorescence microscopic images showing the subcellular localization of *35S::PtaHMGB2/3:YFP::tNOS* (**a**) and *35S::YFP::tNOS* (**b**) in *Nicotiana benthamiana* epidermal cells. *White arrow* indicates nuclear accumulation of PtaHMGB2/3:YFP. *Scale bars* are 50 μm. **c** PtaHMG2/3 mRNA expression levels obtained by qRT-PCR in poplar leaves samples transiently expressing *35S::amiRNA_PtaHMGB2/3::tNOS*–*35S::GFP::tNOS* and transiently expressing the control construct *35S::GFP::tNOS*. Data indicates mean of two independent experiments. **d** Luciferase activity detected in leaf discs when *35S::PtaHMGB2/3::tNOS*–*35S::GFP::tNOS* is transiently expressed in *pPtaLHY2::LUC* genetic background under LD conditions. **e** Strip chart representing the distribution of luciferase values obtained at ZT1 and ZT25, for transiently transformed leaf discs of PtaHMGB2/3 overexpressing and control, respectively. *Horizontal bar* represents the median. This experiment was performed twice. A representative result is shown in *this figure*. **f** Luciferase activity detected in leaf discs when *35S::amiRNA_PtaHMGB2/3::tNOS*–*35S::GFP::tNOS* is transiently expressed in *pPtaLHY2::LUC* genetic background under LD conditions. **g** Strip chart representing the distribution of luciferase values obtained at ZT1 and ZT25, for transiently transformed leaf discs of PtaHMGB2/3 knockdown and control, respectively. *Horizontal bar* represents the median. This experiment was performed twice. A representative result is shown in *this figure*. Control and transiently transformed luciferase measurements were compared with *U*-Mann–Whitney test at ZT1 and ZT25 (**d**, **g**). *P ≤ 0.05; **P ≤ 0.01; ***P ≤ 0.001

To functionally test *PtaHMGB2/3*, firstly we studied its subcellular localization *in planta* by transiently expressing *35S::PtaHMGB2/3:YFP* in *N. benthamiana*. Imaging of PtaHMGB2/3:YFP by fluorescence microscopy showed a nuclear location of the protein (Fig. 3a), while a construct expressing free YFP was detected throughout the whole cell (Fig. 3b). Our result showed that PtaHMGB2/3 has plant nuclear localization fate. In addition to this, we generated a knock down construct using an artificial microRNA (*35S::amiRNA_PtaHMGB2/3*). To study the ability of the amiRNA to knock down the *PtaHMGB2/3* levels, we transiently expressed *35S::amiRNA_PtaHMGB2/3* in poplar leaves using the agroinfiltration method previously described [68]. Our qRT-PCR analysis showed that the expression of *HMGB2/3* decreased approximately in a 50% (Fig. 3c).

To investigate the potential role of *PtaHMGB2/3* upon the expression of our transcriptional reporter, we agroinfiltrated the overexpressing (*p35S::PtaHMGB2/3:tNOS*–*p35* *S*::GFP:tNOS) and the amiRNA constructs (*p35S::amiRNA_HMGB2/3:tNOS*–*p35* *S*::GFP:tNOS) in *pPtaLHY2::LUC* #5 transgenic line. After 3 days post-infiltration, we selected and sectioned GFP expressing agro-infiltrated leaf discs and registered their luciferase activity in a luminometer during 2 days under LD conditions (Additional file 3: Figure S1). The explants that overexpressed the *PtaHMGB2/3* registered higher luciferase values than the control in a gate-dependent manner (Fig. 3d, e). According to that, knocking down *PtaHMGB2/3* decreased luciferase activity at the peak time (Fig. 3f, g). All together these results showed that overexpressing PtaHMGB2/3 not only can increase the activity of *PtaLHY2* promoter, but also that this protein allows *pPtaLHY2::LUC* reporter to reach its proper activity levels.

**Fig. 4:**
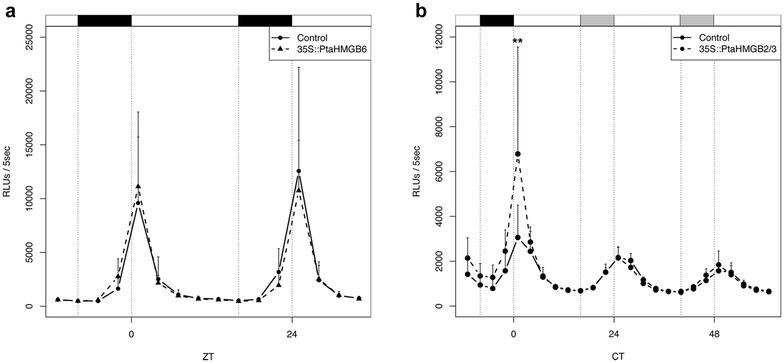
Darkness promotes PtaHMGB2/3 but not PtaHMGB6 activation of *pPtaLHY2::LUC*. **a** Luciferase activity detected in leaf discs when *35S::PtaHMGB6::tNOS*–*35S::GFP::tNOS* is transiently expressed in *pPtaLHY2::LUC* genetic background under LD conditions. This experiment was performed twice. A representative result is shown in this figure. **b** Luciferase activity detected in leaf discs when *35S::PtaHMGB2/3::tNOS*–*35S::GFP::tNOS* is transiently expressed in *pPtaLHY2::LUC* genetic background under LD long days during 24 h and then transferred to LL during the following 48 h. This experiment was performed twice. A representative result is shown in *this figure*

Since many members belonging to HMGB protein family have been described as transcriptional enhancers [45, 53], we tested whether the activation observed in *pPtaLHY2::LUC* is a common ability of HMGB proteins. According to this, we assayed PtaHMGB6 which is phylogenetically distant to PtaHMGB2/3 (Fig. 2). The overexpression of *PtaHMGB6* did not change the expression of *pPtaLHY2::LUC* reporter (Fig. 4a). This absence of activation could be due to PtaHMGB6 did not reach the same protein levels than PtaHMGB2/3. To check this hypothesis, PtaHMGB2/3:YFP, PtaHMGB6:YFP and the free YFP were transiently expressed in poplar leaves. The YFP fluorescence emitted by each transfected discs were measured to estimate protein abundance. We corroborated that both PtaHMGB2/3:YFP and PtaHMGB6:YFP were expressing at similar levels (Additional file 7: Figure S5a). Afterwards, discs showing similar YFP fluorescence, were placed in the 96-well plate to measure luciferase activity and check their functionality upon *pPtaLHY2::LUC* reporter. While the *PtaHMGB2/3:YFP* construct was able to significantly activate *LHY2* promoter activity, no effect was shown when overexpressing *35S::PtaHMGB6:YFP* (Additional file 7: Figure S5b). These results indicate that the enhanced activation of *pPtaLHY2::LUC* observed during PtaHMGB2/3 overexpression is not a common feature of both HMGB proteins, indicating that PtaHMGB2/3 and PtaHMB6 are not redundant in the enhanced activation of *pPtaLHY2::LUC.*

### PtaHMGB2/3 activation of *pPtaLHY2::LUC* is dark-dependent

Our automatic bioluminescence system showed that *pPtaLHY2::LUC* rhythm persists in constant light conditions very likely prompted by the circadian clock (Fig. 1a). To investigate whether overexpression of *PtaHMGB2/3* enhances the activation of *pPtaLHY2::LUC* in constant light conditions, we transiently overexpressed *PtaHMGB2/3* in *pPtaLHY2::LUC* plants and monitored their bioluminescence during 1 day in LD and 2 days in LL conditions. After the first day in LD, we observed the enhanced transcriptional activity of *pPtaLHY2::LUC* reporter (Fig. 3d, e), however the effect vanished in LL conditions (Fig. 4b). These results suggest a critical role of darkness in the proper functioning of PtaHMGB2/3 upon *pPtaLHY2::LUC*.

### PtaHMGB2/3 is diurnally controlled at protein level

**Fig. 5:**
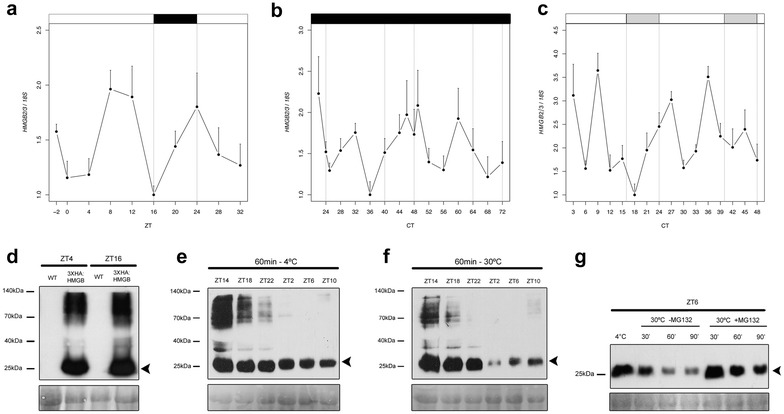
PtaHMGB2/3 protein abundance shows diurnal pattern. **a**–**c** *PtaHMG2/3* mRNA expression profile obtained by qRT-PCR in long days conditions (LD) (**a**), continuous dark (DD) (**b**), and continuous light (LL) (**c**). **d** Immunodetection of 3xHA::PtaHMGB2/3 protein abundance obtained by western blotting. Protein extracts were obtained at ZT4 and ZT16 from wild type and *3xHA:PtaHMGB2/3* expressing *Nicotiana benthamiana* leaves samples grown under LD. **e**, **f** Immunodetection of 3xHA:PtaHMGB2/3 protein abundance obtained by western blotting. *Nicotiana benthamiana* leaves samples expressing 3xHA::PtaHMGB2/3 were collected in a 24 h time course experiment under LD conditions. Protein extracts were incubated in cell free degradation buffer during 60 min at 4 °C (**e**) and 30 °C (**f**). **g** Immunodetection of 3xHA:PtaHMGB2/3 protein abundance by western blot. *Nicotiana benthamiana* leaves samples expressing 3xHA::PtaHMGB2/3 were collected at ZT6 under LD conditions. Protein extracts were incubated in cell free degradation buffer during 30, 60 and 90 min at 30 °C or 90 min at 4 °C, with or without proteasome inhibitor MG132 50 μM. Top labelling indicates Zeitgeber times (ZT) of each protein sample. *Black arrowhead* shows the localization 3xHA:PtaHMGB2/3 in the blot. *Red* ponceau staining of the membrane is shown as loading control. All immunodetection experiments were performed twice. A representative result is shown in *this figure*

The gate-dependent activation of *pPtaLHY2::LUC* suggests that PtaHMGB2/3 activity should be tightly controlled. Previous research carried out in *Pharbitis nil*, demonstrated that the mRNA of an HMG protein has an endogenous rhythm when plants were placed in continuous dark conditions (DD) [43]. To fully characterize *PtaHMGB2/3* gene expression, we carried out time series qRT-PCR analyses of poplar mRNA leaf samples collected in three different conditions (LD, DD, LL) (Fig. 5a–c). The results revealed non-robust rhythm in all tested conditions, indicating that there is neither diurnal nor circadian regulation of *PtaHMGB2/3* mRNA accumulation in poplar, contrasting what was reported for HMG1 of *Pharbitis nil*.

We wondered whether a differential protein accumulation or degradation rate might explain the gate-dependent activation observed when PtaHMGB2/3 is constitutively overexpressed. To investigate PtaHMGB2/3 protein accumulation or degradation *in planta,* we choose *N. benthamiana* agroinfiltration system that has been successfully applied to analyze proteasome-dependent protein degradation as well as ubiquitination in vivo or in vitro reactions [69, 87, 88]. We transiently overexpressed PtaHMGB2/3 tagged with 3xHA epitope in *N. benthamiana* plants and performed western blotting with wild type and 3xHA:PtaHMGB2/3 expressing leaves, collecting the samples at ZT4 and ZT16. The results showed a specifically detection of a band around 25 kDa, which is the expected molecular weight of our fusion protein (Fig. 5d). In addition to this, we specifically detected a high molecular weight track of bands, at both time points assayed, that resemble some kind of protein modification [33, 69].

To carry out PtaHMGB2/3 degradation assay, we transiently overexpressed PtaHMGB2/3 tagged with 3xHA epitope in *N. benthamiana* plants and performed time series analysis on leaf samples collected every 4 h. After that, cell free degradation assays followed by western blot analyses were performed with the protein leaf extracts [33]. This assay permits in vitro enzymatic reactions, such as, proteases and proteasome degradation, in contrast to the previous experiment (Fig. 5d) in which we only can detect protein abundance in a steady state conditions. While in cold treatment the 25 kDa band showed similar abundance in the different time points analyzed (Fig. 5e), at 30 °C we detected the highest degradation of this band at ZT2, a mild degradation at ZT6 and 10, and no degradation from ZT14 to 22 (Fig. 5f). In addition to the 25 kDa band, we observed that the high molecular weight track of bands exhibited a diurnal cycle of protein degradation even at 4 °C, increasing at 30 °C (Fig. 5e, f). Hence, the higher molecular weight version of PtaHMGB2/3 is more susceptible to degradation than the 25 kDa band. These results indicate that the degradation of PtaHMGB2/3 is mostly restricted to the light period.

Finally, to study the potential role of the 26S proteasome in the degradation of PtaHMGB2/3, we performed a cell free degradation assay with the ZT6 protein leaf extract, in the presence and absence of the proteasome inhibitor MG132. While in the absence of MG132 we observed a progressive degradation among the time points assessed, the presence of the drug inhibited this degradation (Fig. 5g). Collectively, these results indicate that the 26S proteasome creates a differential PtaHMGB2/3 protein degradation during the day. This could contribute to the gate-dependent activation of *pPtaLHY2::LUC* observed when *PtaHMGB2/3* is constitutively overexpressed *in planta.*

## Discussion

### Monitoring diurnal and circadian rhythms using stable transcriptional reporters in poplar

Luciferase-based transcriptional reporters to monitor diurnal and circadian rhythms have been widely used in *Arabidopsis*, however their use in poplar has only been shown in poplar transient assays or poplars transformed with well-known *Arabidopsis* circadian reporters [10, 68]. Here, we reported a step-by-step procedure to design and validate the activity of a poplar transcriptional reporter based on the clock gene *PtaLHY2*. Using the *pPtaLHY2::LUC* reporter line, we adapted an automatic luminescence microplate reader for continuous monitoring diurnal and circadian rhythms in poplar tissues, previously used in *Arabidopsis*, green algae and tobacco [33, 89, 90].

Our stable transformed *pPtaLHY2::LUC* reporter line reproduces the *PtaLHY2* mRNA expression pattern in LD and LL. Remarkably, our RAE analysis demonstrated the robustness of the rhythm of our stably reporter poplar lines, which improved the one observed for *pPttLHY::LUC* in transient reporter assays [68]. A plausible explanation for this improved robustness of our poplar reporter would be the fact that the extrachromosomal expression of a transiently transformed *pPttLHY::LUC* reporter is not under chromatin regulation. Indeed, an increasing number of publications have pointed to the importance of the chromatin organization in fine tuning gene expression, particularly in plant clock genes [17, 91–93]. Therefore, stably integrated transcriptional reporter in poplar plants is desirably when robustness of rhythms is required, or even more, when chromatin regulation levels are considered for further analysis. However, *cis*-regulatory elements and promoter sequences are essential to generate the rhythms. Therefore, the use of diurnal and circadian transcriptional reporters to analyse rhythms in transient assay are very useful to identify *cis*-regulatory elements in loss of function analyses or to study the activity of transcriptional factors by transactivation assays [94–96].

### Using poplar stable transcriptional reporters to functionally study new regulators of diurnal and circadian rhythms

The study of transcriptional regulation in herbaceous plants mainly relies on forward genetic screening, followed by an extensive molecular analysis of the candidate gene function. However, in perennial plants, the scarce available information about transcriptional regulation mainly comes from functional studies of selected transcription factors in poplar species, which require spending long time generating transgenic plants. We set up a system for rapid evaluation of the transcriptional regulatory function of candidate genes in a temporal manner, using a transcriptional reporter as a genetic background. We applied the transient expression methodology, developed by Takata and Eriksson [68], to transiently overexpressing and knocking down a candidate gene. Using an automatic luminescence microplate reader, the potential alteration of the reporter was examined in individual selected leaf discs for two consecutive days.

In this work, we used this transactivation assay in poplar as a quick approach to investigate the potential role of a protein as a diurnal and circadian regulator of the biological clock, using our *pPtaLHY2::LUC* transcriptional reporter. Moreover, this system could be used to rapidly assess in poplar the transcriptional regulation of other proteins upon non-circadian reporter targets, under biotic or abiotic stresses in a temporal manner. Similar strategy has been applied to unravel the consequences of the interaction between *Arabidopsis* proteins PIF4 and ELF3 upon the *Arabidopsis PIL1* promoter in tobacco leaves [89]. Transactivation studies have also been performed to demonstrate the role of the interaction between poplar FT and FD upon the activation of *OsMADS15* in rice protoplasts [4]. Moreover, transgenic poplars carrying Like-APETALA1:GR construct were used to demonstrate its role as activator of *AINTEGUMENTA*-*like1* (*AIL1*) [3]. In a similar manner, poplar trees which overexpressed *PHYA* or *FT*, were used to observed changes in *AIL1* [2]. All these experiments could have been performed directly in poplar without the requirement of generating a double transgenic plant.

We would like to draw attention, however, to the high difference regarding transformation efficiency when comparing this protocol to other transient methodologies followed in *Arabidopsis* [97] or tobacco [69]. Working with the latest protocols, almost every leaf in the plant is uniformly transfected. Nevertheless, using the protocol with poplar, we tend to observe isolated leaf patches successfully transfected. To deal with this pitfall, we increased the number of plants that had to be transfected in every experiment. Moreover, we included the *35S::GFP:tNOS* cassette in the binary vectors along with our potential regulators, which allowed us to select those leaf areas that were successfully transfected.

### Participation of HMGB proteins in controlling diurnal and circadian rhythms

Using this methodology, we study the role of two representative members of poplar HMGB proteins revealing a novel function for this type of nuclear regulators. There is an extensive biochemical characterization of HMGB proteins in plants, which has allowed to define their transcriptional regulatory activity as chromatin remodelers and chaperones, facilitating the entrance and binding of specific transcription factors to DNA [40, 41, 48, 98, 99]. Nevertheless, only a few works have been published describing their participation in particular biological processes [100, 101], none of them in poplar.

By using our transient transactivation assay in poplar, we demonstrated that PtaHMGB2/3, but not PtaHMGB6, promotes the activity of *PtaLHY2* promoter in a gate-dependent manner. Moreover, its activity is observed only under LD conditions, particularly at dawn. PtaHMGB2/3 and PtaHMGB6 present their HMG-box domains mostly conserved, although they have large amino acid sequence differences in their characteristic basic N-terminal and acidic C-terminal. HMG proteins bind DNA by their HMG-box domain and by their acidic C-terminal [45, 46]. Furthermore, HMG-box mediates the interaction with other proteins [48, 102]. The differential ability of PtaHMGB2/3 and PtaHMGB6 upon the activity of *PtaLHY2* promoter may be explained by a differential DNA affinity. However, due to the dissimilarities found in their primary protein structure, we cannot exclude the possibility of the interaction with other transcription factor. In fact, this possibility might also explain why the PtaHMGB2/3 needs darkness to promote the activation of *PtaLHY2* promoter.

Remarkably, our transient transactivation assays also revealed that the gate-dependent activation of *pPtaLHY2::LUC* is produced under a constitutive expression of PtaHMGB2/3. Two reasons could explain this marked temporal restriction of PtaHMGB2/3 activity: (1) PtaHMGB2/3 is posttranslationally controlled showing a diurnal pattern that overlaps with *PtaLHY2* activation and/or (2) PtaHMGB2/3 activity depends on a diurnally controlled factor.

Even though *PtaHMGB2/3* mRNA accumulation clearly showed no robust oscillatory mRNA pattern under LD, LL and DD conditions, we found that PtaHMGB2/3 protein has a marked degradation rate depending on the time of the day, and that this degradation is mediated by the 26S proteasome. This is a novel feature for HMGB proteins in plants that is in accordance with the circadian regulation of protein accumulation reported for Hmgb1 in rat retinal photoreceptor cells. Rat Hmgb1 presents a diurnal oscillation with its peak of abundance around ZT6 and its trough at midnight [42]. The daily pattern of protein stability in these HMGBs, suggests a higher level of specialization in their function. In this work, we showed that *PtaLHY2* transcription starts rising from midnight, so presumably proteins that influence its expression should be present at that time. Hence, this specific control of protein stability could contribute to the gate-dependent activation of *pPtaLHY2::LUC* by PtaHMGB2/3.

The high molecular weight bands that we specifically detected in the immunoblots, present at ZT4 and ZT16 in the protein abundance assay, indicated their potential posttranslational modification is not diurnally controlled. However, in the protein degradation experiment, they showed a marked diurnal pattern, even at 4 °C. Moreover, the abundance of this high molecular weight track of bands was more susceptible to degradation than the 25 kDa band. HMG proteins can be posttranslationally modified with acetylation, methylation, phosphorylation and ubiquitination [40, 41, 48, 54, 98, 99, 103]. These modifications control the import and export to and from the nucleus, respectively [54] but also regulate the binding to DNA [40, 99] and the interaction with other proteins [48, 102]. Therefore, these high molecular weight versions of PtaHMGB2/3 might contribute to control its activity, perhaps affecting the protein stability. Nevertheless, their biological role needs to be further study.

Although we provide evidences that PtaHMGB2/3 is posttranslationally modified, we cannot exclude that PtaHMGB2/3 activity depends on a diurnally controlled factor. Previous work demonstrated that HMGB proteins interact with transcription factors improving their binding to DNA [45, 48]. One plausible scenario could be that HMGB2/3 interacts with a photosensitive transcription factor. Thus, the presence of this factor during the night would provide competence to PtaHMGB2/3 to promote the gate-dependent activation of *pPtaLHY2::LUC.* This hypothesis might also shed light on why the amplitude of *pPtaLHY2::LUC* in LL is much lower than in LD. Future work will evaluate this hypothesis.

## Conclusion

In this work, we generated a stable luciferase reporter line based on the *PtaLHY2* clock gene. Using this reporter line as a genetic background, we established a methodology to rapidly assess potential regulators of diurnal and circadian rhythms. This procedure allowed us to demonstrate that PtaHMGB2/3 promotes the transcriptional activation of *PtaLHY2* circadian reporter in a gate-dependent manner. Moreover, we added new information about the diurnal control of the PtaHMGB2/3.

## Additional files

**Additional file 1: Table S1.** Primers used in this work.

**Additional file 2: Table S2.** Description of GoldenBraid parts used in this work.

**Additional file 3: Figure S1.** Detailed steps of the microplate preparation previous to luminescence measurement. **a** Scheme of the construct created to transiently co-express the amiRNA_PtaHMG2/3 and GFP reporter gene in poplar leaf cells. Similar strategy was followed for the rest of proteins used in this work. **b** Fluorescence image showing a poplar leaf agroinfiltrated with the construct represented in a. GFP fluorescent leaf disc is cut from GFP expressing leaf patches using a hole puncher. **c** Selected leaf disc are placed in a 96-well microplate containing solid MS1B agar without sucrose and with D-Luciferin substrate.

**Additional file 4: Figure S2.** Molecular Phylogenetic analysis by Maximum Likelihood method. **a** The evolutionary history was inferred by using the Maximum Likelihood method based on the JTT matrix-based model. The tree with the highest log likelihood (-4000.0116) is shown. The percentage of trees in which the associated taxa clustered together is shown next to the branches. Initial tree(s) for the heuristic search were obtained automatically by applying Neighbor-Join and BioNJ algorithms to a matrix of pairwise distances estimated using a JTT model, and then selecting the topology with superior log likelihood value. The rate variation model allowed for some sites to be evolutionarily invariable ([+ I], 27.6154% sites). The tree is drawn to scale, with branch lengths measured in the number of substitutions per site. The analysis involved 8 amino acid sequences. All positions containing gaps and missing data were eliminated. There were a total of 748 positions in the final dataset. **b**, **c** *pPtaLHY2::LUC* activity detected by luminescence assay during 2 days under LD and 2 days under LL conditions. Line #7 is shown in (**b**) and line #10 is shown in (**c**). Data indicates mean of three independent assays. Error bars indicate SD of the mean. **d** *PtaLHY2* mRNA expression profile in LL condition obtained by qRT-PCR. Data indicates mean ± SD of three technical replicates. This experiment was performed twice. A representative result is shown in this figure. Upper bar indicates the photoperiod: white boxes indicate day and grey boxes indicate subjective night.

**Additional file 5: Figure S3.** Molecular Phylogenetic analysis of HMGB family by Maximum likelihood and UPGMA methods. **a** The evolutionary history was inferred by using the Maximum Likelihood method based on the Whelan And Goldman model. The tree with the highest log likelihood (-2313.3530) is shown. The percentage of trees in which the associated taxa clustered together is shown next to the branches. Initial tree(s) for the heuristic search were obtained automatically by applying Neighbor-Join and BioNJ algorithms to a matrix of pairwise distances estimated using a JTT model, and then selecting the topology with superior log likelihood value. A discrete Gamma distribution was used to model evolutionary rate differences among sites (3 categories (+G, parameter = 2.7456)). The tree is drawn to scale, with branch lengths measured in the number of substitutions per site. The analysis involved 15 amino acid sequences. All positions containing gaps and missing data were eliminated. There were a total of 113 positions in the final dataset.** b** The evolutionary history was inferred using the UPGMA method. The optimal tree with the sum of branch length = 4.036 is shown. The percentage of replicate trees in which the associated taxa clustered together in the bootstrap test (500 replicates) is shown next to the branches. The tree is drawn to scale, with branch lengths in the same units as those of the evolutionary distances used to infer the phylogenetic tree. The evolutionary distances were computed using the Poisson correction method and are in the units of the number of amino acid substitutions per site. The analysis involved 15 amino acid sequences. All positions containing gaps and missing data were eliminated. There were a total of 113 positions in the final dataset.

**Additional file 6: Figure S4.** Pairwise alignment of PtaHMGB2/3 and PtaHMGB6 protein sequences. PtaHMGB2/3 and PtaHMGB6 protein sequences alignment obtained by MUSCLE aligning tool and decorated using BioEdit software. Residues shaded in black are identical. Residues shaded in gray denote conserved substitutions. Amino acids belonging to HMG-box domain have been highlighted with a red box. Basic and acidic tails has been highlighted with a blue and green line, respectively.

**Additional file 7: Figure S5.** Determination of PtaHMGB2/3 and PtaHMGB6 protein abundance followed by *pPtaLHY2::LUC* reporter assay. **a** Fluorescence of YFP and PtaHMGB2/3:YFP and PtaHMGB6:YFP fusion proteins was quantified in each poplar leaf discs. *pPtaLHY2::LUC* leaf discs were used to set the fluorescence background. The boxplot represents the distribution of the relative fluorescence (fold increase) values normalized against the fluorescence background of all discs used in the experiments (two biological replicates). The black horizontal line indicates the median. Different letters indicate statistical differences assessed by One Way ANOVA (F_3,36_ = 22.59, p < 0.001) and Tukey test (α = 0.05). Scale bar = 1.5 mm. **b** Luciferase values of pPtaLHY2::LUC reporter line #5 discs transfected with *35S::YFP* (control), *35S::PtaHMGB2/3:YFP* or *35S::PtaHMGB6*. All discs whose fluorescence was previously quantify were place to measure luciferase activity. This experiment was repeated twice. From every replicate, we calculated a single mean from the RLU values of every disc involved in the experiment. Values in this plot indicate the average between the mean values obtained for each replicate. Error bars indicate SEM (n = 2 biological replicates). Different letters indicate statistical differences assessed by One Way ANOVA at ZT1 (F_2,3_ = 45.22, p < 0.01) and Dunnet’s test (p_YFP vs. HMGB2/3:YFP_ < 0.01; p_YFP vs. HMGB6:YFP_ > 0.05).

## Abbreviations

*LHY2*: late elongated hypocotyl 2
HMG: high mobility group
YFP: yellow fluorescence protein
GFP: green fluorescence protein
LUC: luciferase
LD: long days
LL: continuous light
DD: continuous dark
ZT: zeitgeber time
CT: circadian time
RAE: relative amplitude error
