# Supplementary material for: Real-time monitoring of PtaHMGB activity in poplar transactivation assays
Source: Plant Methods. 2017 Jun 15;13:50. doi: 10.1186/s13007-017-0199-x (PMC5472981; doi:10.1186/s13007-017-0199-x)
Supplement: Supplementary file 1 — Additional file 1: Table S1. Primers used in this work. [file 13007_2017_199_MOESM1_ESM.docx]

**Table S1.** Ramos-Sánchez et al.

| Primer name | Sequence (5’ → 3’) | Annotation |
| --- | --- | --- |
| pPtaLHY2_fwd | CATATCACAATCTATCATTACTCATCGTTG | To amplify PtaLHY2 promoter |
| pPtaLHY2_rev | TTTACCTTAATAACCAAGTCTTCCCCAG |  |
| pPtaLHY2_fwd_GW | GGGGACAAGTTTGTACAAAAAAGCAGGC  TTACCGTATAGAACCGATCCTTCTCA | To clon PtaLHY2 promoter into pDONR207 |
| pPtaLHY2_rev_GW | GGGGACCACTTTGTACAAGAAAGCTGG  GTCTAGCGGACCTTAGGCAGCCAACG |  |
| PtaHMGB2/3_fwd_ AATGprefix | GCGCCGTCTCGCTCGAATGAAAGGAGGT  AGATCAAAGTC | To clon PtaHMGB2/3 into pUPD domestication vector – overexpression construct |
| PtaHMGB2/3_rev_  GCTTsuffix | GCGCCGTCTCGCTCGAAGCCTAACAAGAT  GCACCAGCTC |  |
| PtaHMGB2/3_fwd_ AGCCprefix | GCGCCGTCTCGCTCGAGCCATGAAAGGA  GGTAGATCAAAGTCAGATACC | To clon PtaHMGB2/3 into pUPD domestication vector – 3xHA fusion |
| PtaHMGB2/3_fwd_GCAGsuffix | GCGCCGTCTCGCTCACTGCCTAACAAGATGCACCAGCTC | To clon PtaHMGB2/3 into pUPD domestication vector – YFP fusion |
| PtaHMGB6_fwd_ AATGprefix | GCGCCGTCTCGCTCGAATGGCAAGGAA  GAGAGTGGA | To clon PtaHMGB6 into pUPD domestication vector – overexpression construct |
| PtaHMGB6_rev_  GCTTsuffix | GCGCCGTCTCGCTCGAAGCTCACAGTT  CATGATCAGCTAC |  |
| PtaHMGB6_rev_  GCAGsuffix | GCGCCGTCTCGCTCGCTGCCAGTTCATGATCAGCTACTTC | To clon PtaHMGB6into pUPD domestication vector – YFP fusion |
| 35S_primer | CCCACTATCCTTCGCAAGACC | To generate amiRNA_PtaHMGB2/3 construct as described in Shi et al., (2010) |
| tNOS_primer | TTTATTGCCAAATGTTTGAACGATC |  |
| PtaHMGB2/3_miR-s_1P | CCTAAAAATGCGCTAGCACGTTTTGTGG  CTCTTCCTTTTC |  |
| PtaHMGB2/3_miR-a_2P | AAAAGCGTGCTAGCGCATTTTTAGGGTA  GAGCCAAAACAAG |  |
| PtaHMGB2/3_miR*-s_3P | AAAAACGTGCTAGCGGATTTTTTGGATG  GAGCTACTAACAG |  |
| PtaHMGB2/3_miR*-a_4P | CCAAAAAATCCGCTAGCACGTTTTTCAT  CTGTCTCTGCTCC |  |
| amiRNA408_pUPD_  fwd_AATGprefix | GCGCCGTCTCGCTCGAATGCAAGGGGA  AGCGTGTTCCAA | To adapt amiRNA408 to GoldenBraid cloning system. |
| amiRNA408_pUPD_  rev_GCTTsuffix | GCGCCGTCTCGCTCGAAGCTGTCCTCAA  AGGACGTTTGTC |  |
| qPCR_PtaHMGB2/3_  fwd | CAAGGCTGATAAAAGGAAAGTC | For qPCR analysis |
| qPCR_PtaHMGB2/3_  rev | ACAAGATGCACCAGCTCTAAC |  |
| qPCR_PtaLHY2_fwd | GAGGTTTCCAATCCAGGCAA |  |
| qPCR_PtaLHY2_rev | GGCAGGCAAACGAGGTATG |  |
| qPCR_18S_fwd | TCAACTTTCGATGGTAGGATAGTG |  |
| qPCR_18S_rev | CCGTGTCAGGATTGGGTAATTT |  |
