# Supplementary material for: Real-time monitoring of PtaHMGB activity in poplar transactivation assays
Source: Plant Methods. 2017 Jun 15;13:50. doi: 10.1186/s13007-017-0199-x (PMC5472981; doi:10.1186/s13007-017-0199-x)
Supplement: Supplementary file 2 — Additional file 2: Table S2. Description of GoldenBraid parts used in this work. [file 13007_2017_199_MOESM2_ESM.docx]

**Table S2.** Ramos-Sánchez et al.

| Golden Braid reference | Contruct Name | Prefix | Suffix |
| --- | --- | --- | --- |
| pUPD | pUPD | ---- | ---- |
| pDGB1_alpha1 | pDGB1_alpha1 | ---- | ---- |
| pDGB1_alpha2 | pDGB1_alpha2 | ---- | ---- |
| pDGB2_omega1 | pDGB2_omega1 | ---- | ---- |
| GB0359 | 1_alpha1_35S::GFP::tNOS | ---- | ---- |
| GB0030 | p35S | GGAG | AATG |
| GB0037 | tNOS | GCTT | CGCT |
| GB0053 | YFP | AATG | GCTT |
| GB0024 | YFP-Ct | GCAG | GCTT |
| GB0251 | P3xHA | AATG | AGCC |
| *Made for this work* | pUPD_PtaHMGB2/3 | AATG | GCTT |
| *Made for this work* | pUPD_PtaHMGB2/3_ CtermFusion | AATG | GCAG |
| *Made for this work* | pUPD_PtaHMGB6 | AATG | GCTT |
| *Made for this work* | pUPD_PtaHMGB6_ CtermFusion | AATG | GCAG |
| *Made for this work* | 1_alpha2_35S:: PtaHMGB2/3::tNOS | ---- | ---- |
| *Made for this work* | 1_alpha2_35S:: PtaHMGB2/3:YFP::tNOS | ---- | ---- |
| *Made for this work* | 1_alpha1_35S::3xHA:PtaHMGB2/3::tNOS | ---- | ---- |
| *Made for this work* | 1_alpha2_35S::amiRNA_PtaHMGB2/3::tNOS | ---- | ---- |
| *Made for this work* | 1_alpha2_35S::PtaHMGB6::tNOS | ---- | ---- |
| *Made for this work* | 2_omega1_35S::PtaHMGB2/3::tNOS_ 35S::GFP::tNOS | ---- | ---- |
| *Made for this work* | 2_omega1_35S::amiRNA_PtaHMGB2/3::tNOS_ 35S::GFP::tNOS | ---- | ---- |
| *Made for this work* | 2_omega1_35S::PtaHMGB6::tNOS_ 35S::GFP::tNOS | ---- | ---- |
