# Supplementary figures and images for: Real-time monitoring of PtaHMGB activity in poplar transactivation assays
Source: Plant Methods. 2017 Jun 15;13:50. doi: 10.1186/s13007-017-0199-x (PMC5472981; doi:10.1186/s13007-017-0199-x)

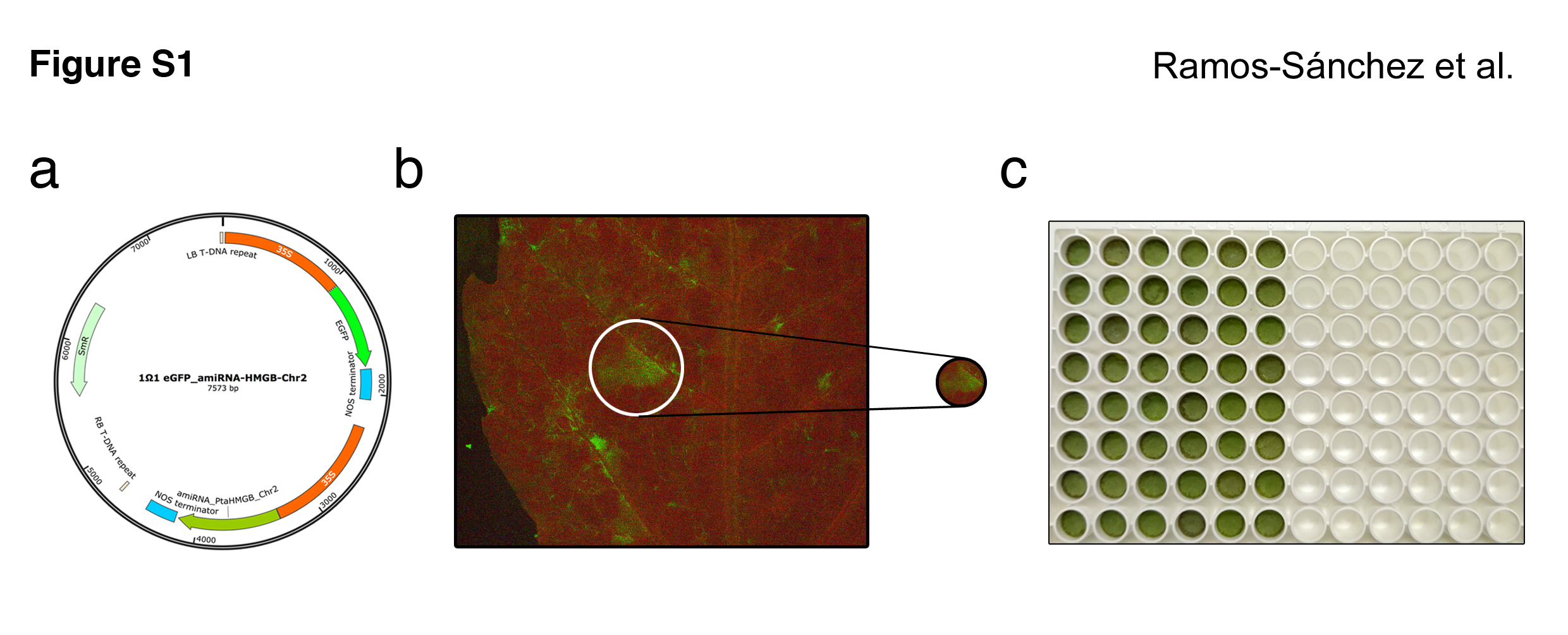

Supplement: Supplementary file 3 — Additional file 3: Figure S1. Detailed steps of the microplate preparation previous to luminescence measurement. a Scheme of the construct created to transiently co-express the amiRNA_PtaHMG2/3 and GFP reporter gene in poplar leaf cells. Similar strategy was followed for the rest of proteins used in this work. b Fluorescence image showing a poplar leaf agroinfiltrated with the construct represented in a. GFP fluorescent leaf disc is cut from GFP expressing leaf patches using a hole puncher. c Selected leaf disc are placed in a 96-well microplate containing solid MS1B agar without sucrose and with D-Luciferin substrate. [file 13007_2017_199_MOESM3_ESM.tif]

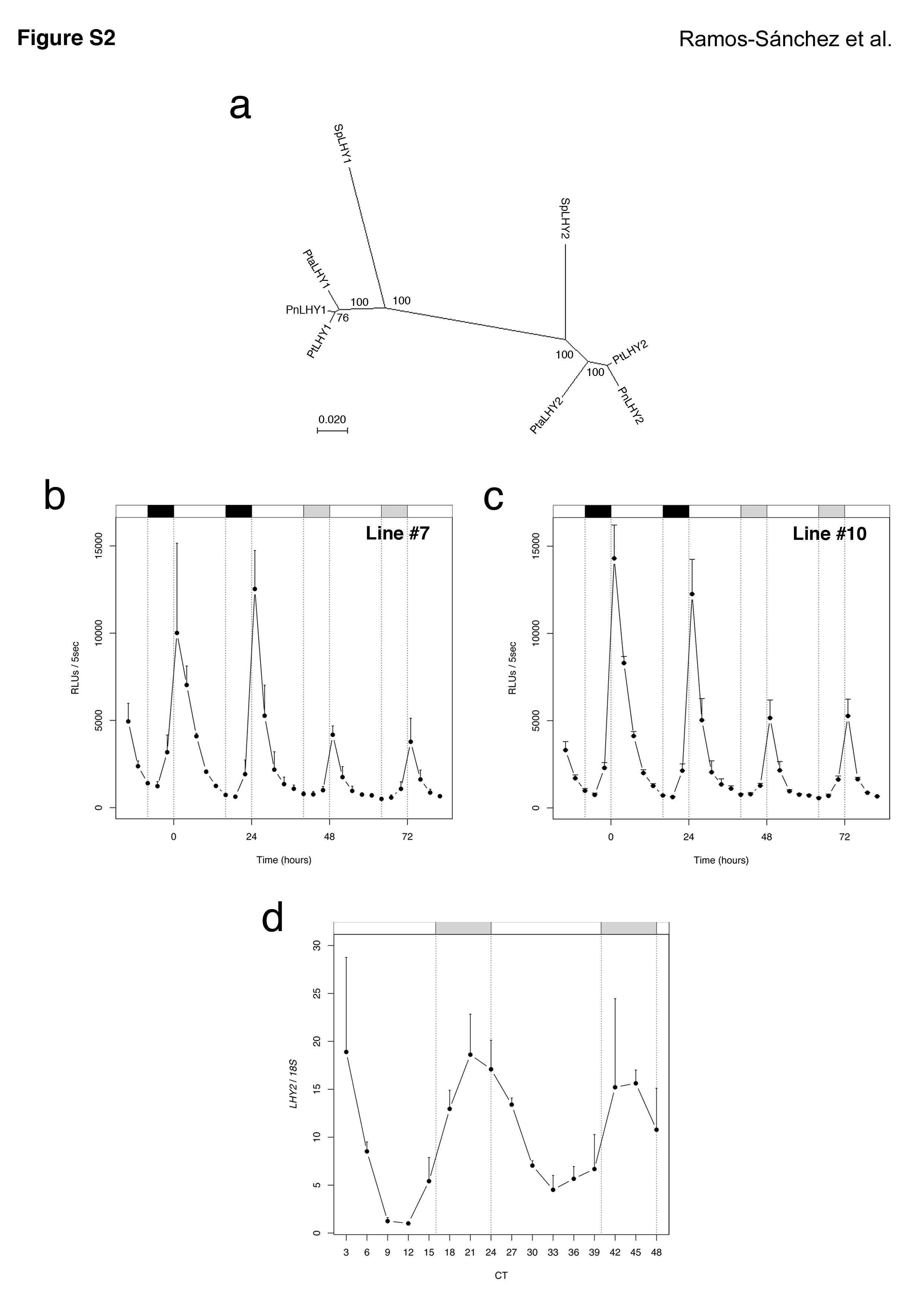

Supplement: Supplementary file 4 — Additional file 4: Figure S2. Molecular Phylogenetic analysis by Maximum Likelihood method. a The evolutionary history was inferred by using the Maximum Likelihood method based on the JTT matrix-based model. The tree with the highest log likelihood (-4000.0116) is shown. The percentage of trees in which the associated taxa clustered together is shown next to the branches. Initial tree(s) for the heuristic search were obtained automatically by applying Neighbor-Join and BioNJ algorithms to a matrix of pairwise distances estimated using a JTT model, and then selecting the topology with superior log likelihood value. The rate variation model allowed for some sites to be evolutionarily invariable ([+ I], 27.6154% sites). The tree is drawn to scale, with branch lengths measured in the number of substitutions per site. The analysis involved 8 amino acid sequences. All positions containing gaps and missing data were eliminated. There were a total of 748 positions in the final dataset. b, c pPtaLHY2::LUC activity detected by luminescence assay during 2 days under LD and 2 days under LL conditions. Line #7 is shown in (b) and line #10 is shown in (c). Data indicates mean of three independent assays. Error bars indicate SD of the mean. d PtaLHY2 mRNA expression profile in LL condition obtained by qRT-PCR. Data indicates mean ± SD of three technical replicates. This experiment was performed twice. A representative result is shown in this figure. Upper bar indicates the photoperiod: white boxes indicate day and grey boxes indicate subjective night. [file 13007_2017_199_MOESM4_ESM.tif]

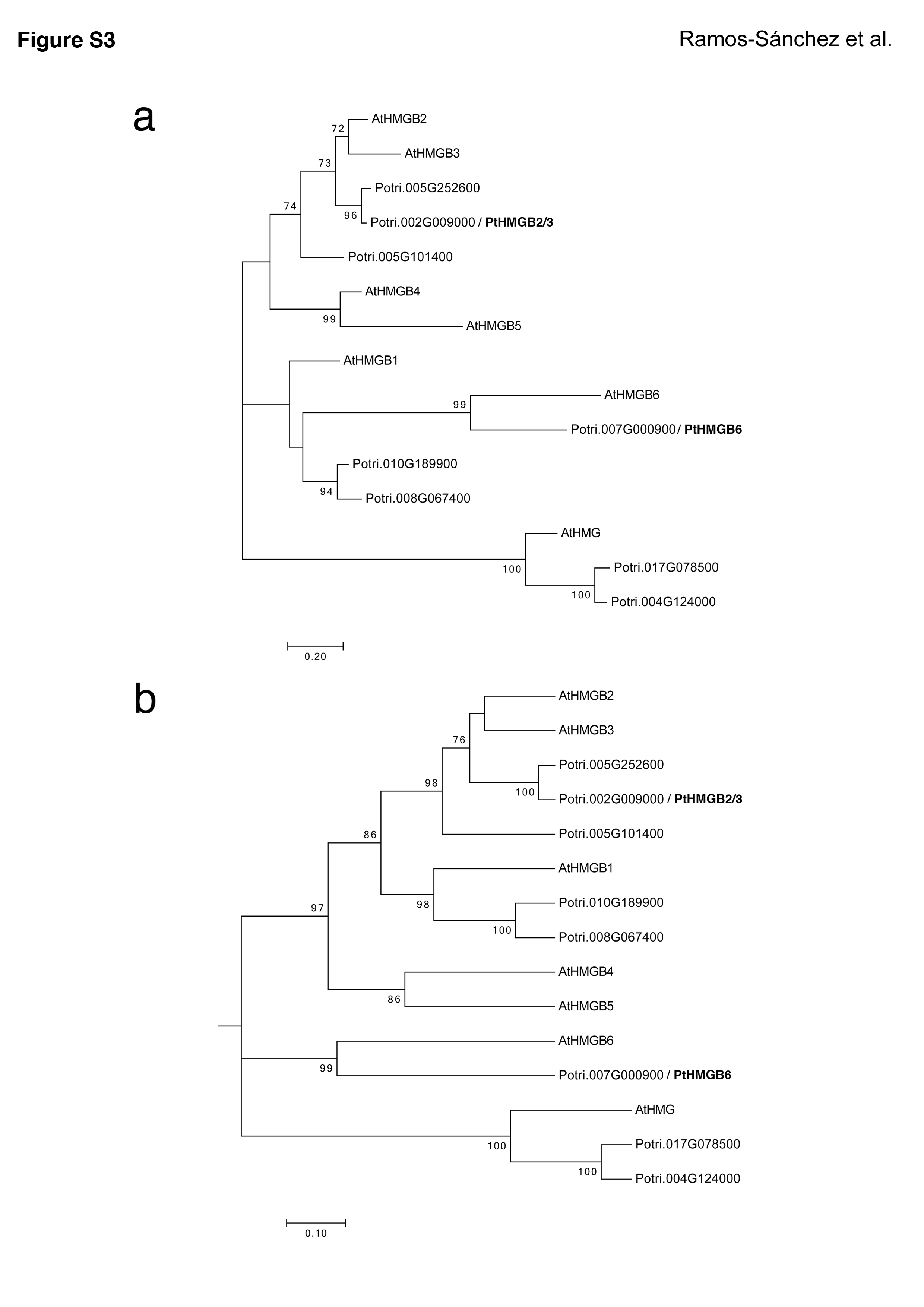

Supplement: Supplementary file 5 — Additional file 5: Figure S3. Molecular Phylogenetic analysis of HMGB family by Maximum likelihood and UPGMA methods. a The evolutionary history was inferred by using the Maximum Likelihood method based on the Whelan And Goldman model. The tree with the highest log likelihood (-2313.3530) is shown. The percentage of trees in which the associated taxa clustered together is shown next to the branches. Initial tree(s) for the heuristic search were obtained automatically by applying Neighbor-Join and BioNJ algorithms to a matrix of pairwise distances estimated using a JTT model, and then selecting the topology with superior log likelihood value. A discrete Gamma distribution was used to model evolutionary rate differences among sites (3 categories (+G, parameter = 2.7456)). The tree is drawn to scale, with branch lengths measured in the number of substitutions per site. The analysis involved 15 amino acid sequences. All positions containing gaps and missing data were eliminated. There were a total of 113 positions in the final dataset. b The evolutionary history was inferred using the UPGMA method. The optimal tree with the sum of branch length = 4.036 is shown. The percentage of replicate trees in which the associated taxa clustered together in the bootstrap test (500 replicates) is shown next to the branches. The tree is drawn to scale, with branch lengths in the same units as those of the evolutionary distances used to infer the phylogenetic tree. The evolutionary distances were computed using the Poisson correction method and are in the units of the number of amino acid substitutions per site. The analysis involved 15 amino acid sequences. All positions containing gaps and missing data were eliminated. There were a total of 113 positions in the final dataset. [file 13007_2017_199_MOESM5_ESM.tif]

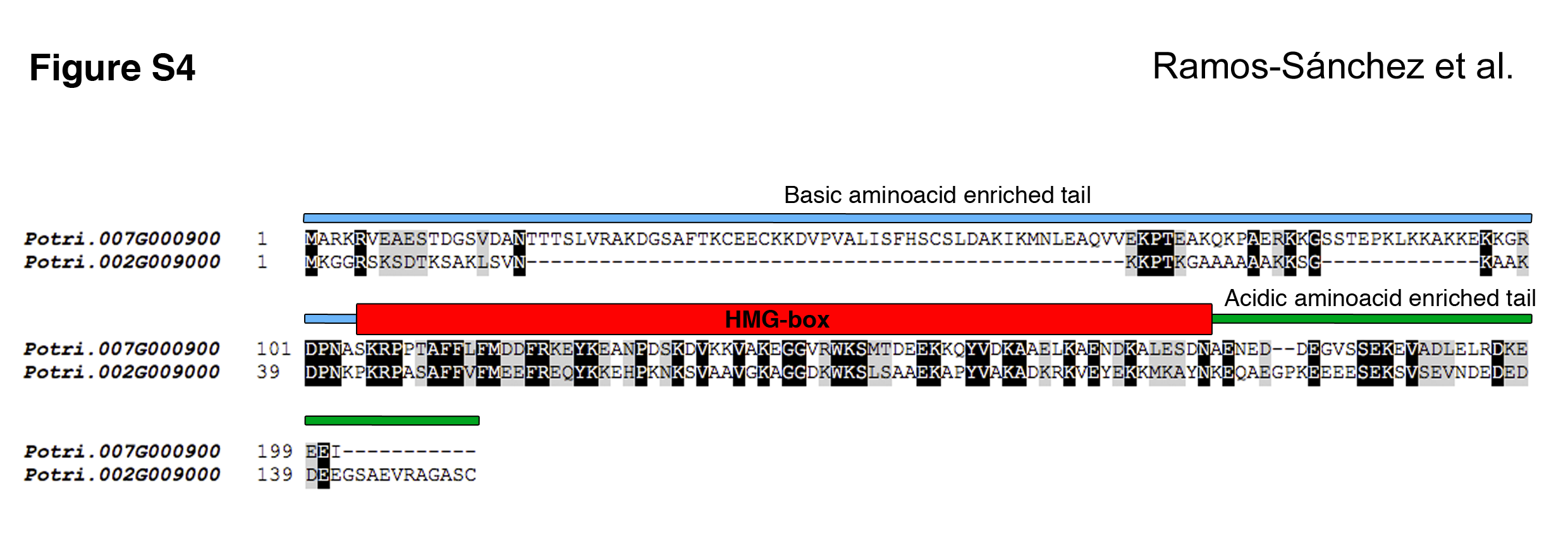

Supplement: Supplementary file 6 — Additional file 6: Figure S4. Pairwise alignment of PtaHMGB2/3 and PtaHMGB6 protein sequences. PtaHMGB2/3 and PtaHMGB6 protein sequences alignment obtained by MUSCLE aligning tool and decorated using BioEdit software. Residues shaded in black are identical. Residues shaded in gray denote conserved substitutions. Amino acids belonging to HMG-box domain have been highlighted with a red box. Basic and acidic tails has been highlighted with a blue and green line, respectively. [file 13007_2017_199_MOESM6_ESM.tif]

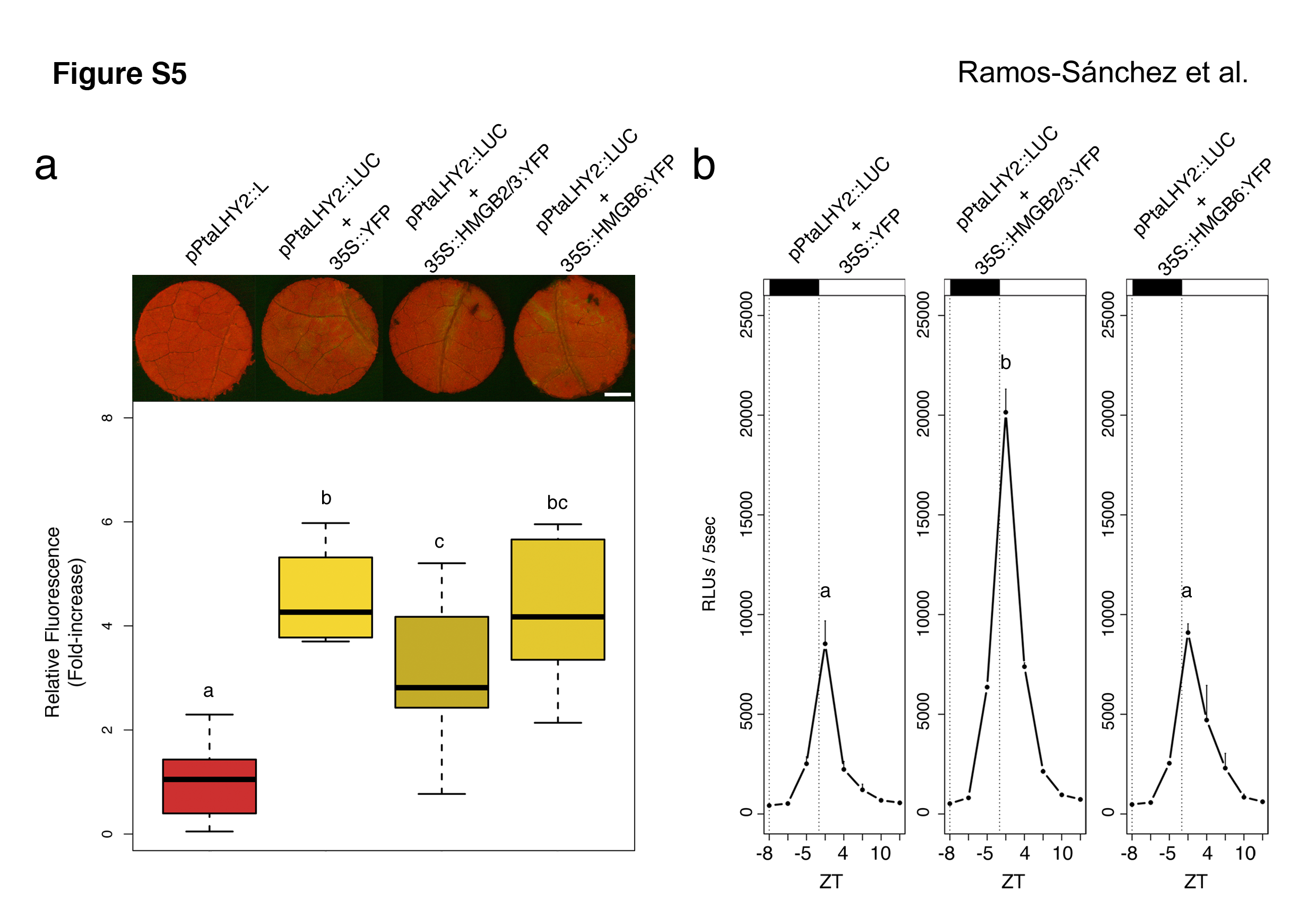

Supplement: Supplementary file 7 — Additional file 7: Figure S5. Determination of PtaHMGB2/3 and PtaHMGB6 protein abundance followed by pPtaLHY2::LUC reporter assay. a Fluorescence of YFP and PtaHMGB2/3:YFP and PtaHMGB6:YFP fusion proteins was quantified in each poplar leaf discs. pPtaLHY2::LUC leaf discs were used to set the fluorescence background. The boxplot represents the distribution of the relative fluorescence (fold increase) values normalized against the fluorescence background of all discs used in the experiments (two biological replicates). The black horizontal line indicates the median. Different letters indicate statistical differences assessed by One Way ANOVA (F3,36 = 22.59, p < 0.001) and Tukey test (α = 0.05). Scale bar = 1.5 mm. b Luciferase values of pPtaLHY2::LUC reporter line #5 discs transfected with 35S::YFP (control), 35S::PtaHMGB2/3:YFP or 35S::PtaHMGB6. All discs whose fluorescence was previously quantify were place to measure luciferase activity. This experiment was repeated twice. From every replicate, we calculated a single mean from the RLU values of every disc involved in the experiment. Values in this plot indicate the average between the mean values obtained for each replicate. Error bars indicate SEM (n = 2 biological replicates). Different letters indicate statistical differences assessed by One Way ANOVA at ZT1 (F2,3 = 45.22, p < 0.01) and Dunnet’s test (pYFP vs. HMGB2/3:YFP < 0.01; pYFP vs. HMGB6:YFP > 0.05). [file 13007_2017_199_MOESM7_ESM.tif]
